# A survey of surgeons’ perception and awareness of intraoperative time utilization

**DOI:** 10.1186/1754-9493-8-30

**Published:** 2014-07-01

**Authors:** Sofia Erestam, Annette Erichsen, Kristoffer Derwinger, Karl Kodeda

**Affiliations:** 1 Institute of Health and Care Sciences, Sahlgrenska University Hospital, Campus Östra, Gothenburg, Sweden; 2 Department of Anaesthesia, Surgery, and Intensive Care, Sahlgrenska University Hospital, Campus Östra, Gothenburg, Sweden; 3 Department of Surgery, Institute of Clinical Sciences, Sahlgrenska Academy at University of Gothenburg, Campus Östra, Gothenburg, Sweden; 4 Department of Colorectal Surgery, Sahlgrenska University Hospital, Campus Östra, Gothenburg, Sweden

**Keywords:** Patient safety, Surgical time, Surgical team, Communication, Operating theatre

## Abstract

**Background:**

Surgical teams’ awareness of the time needed to perform specific phases of a surgical procedure is likely to improve communication in the operating theatre and benefit patient safety. The aim of this study was to assess surgeons’ awareness of time utilization and the actual time needed to perform specific phases of an operation.

**Methods:**

A survey was conducted to examine the method and design for a larger study. Interviews were conducted with 18 surgeons, and surgical time was measured during 21 colon cancer resections. Correlation analyses were performed to explore the factors that might affect operating time.

**Results:**

The surgical phase with the greatest variation in time was dissection/resection (43–308 minutes). On a group level, no statistically significant differences were found between estimated and measured surgical procedural times for partial or full resections (160.4 versus 173.0 minutes, p = 0.539). However, interindividual variation was substantial. There was a positive significant correlation between long duration of dissection/resection and longer time to close the abdomen (r = 0.464, p = 0.039), as well as between long duration of a hand-sewn anastomosis and time needed to close the abdomen (r = 0.536, p = 0.018).

**Conclusions:**

It can be difficult for a single surgeon to estimate the time required for a partial or full surgical procedure. A larger study might provide additional time estimates and identify variables that affect surgical time. The data could be of interest in the planning and scheduling of surgical resources, thus improving theatre team communication and patient safety.

## Background

Surgical procedures in operating theatres (OTs) are complex, high-risk situations that require extensive coordination among individuals and teams [1]. When the surgical programme is too extensive in relation to the present workforce or the OTs available, which often occurs with emergency procedures, a reprioritisation of the operating programme becomes necessary and may lead to cancellations of procedures or to OT staff being forced to work overtime. Variability in the time required for surgical procedures can complicate surgical scheduling and reduce operational efficiency [2].

The time needed to perform defined surgical phases of a colorectal cancer resection has not been well described. A lack of knowledge regarding the time distribution of an operation and a lack of awareness on the part of surgeons regarding actual surgical time can complicate communications regarding the surgical day’s schedule and lead to unnecessary intraoperative interruptions. Thus, it is important to ascertain the actual time required to perform specific phases of a surgical procedure, as well as to examine the factors affecting the duration of an operation.

Surgeons often become involved in intraoperative planning decisions and changing priorities in the surgical schedule. However, it can be difficult for a single surgeon to estimate remaining surgical time, which can lead to inappropriate interruptions during surgery. Patient safety in the OT can be adversely affected by intraoperative interruptions and lack of communication [1,3,4]; OT efficiency, quality of care, and patient safety rely on a high standard of communication and teamwork [5,6].

Awareness of the time needed to perform specific parts of a surgical procedure is thus likely to improve communication in the OT and benefit patient safety. As such, a survey was conducted to assess surgeons’ time use awareness and to determine the actual time needed to perform specific phases of an operation.

## Methods

A pilot study was conducted to test the method and design of a full-scale study whose aim is to address the hypothesis “there is a difference in the estimated and actual surgical time required for specified surgical phases”.

The department where the study was conducted is part of an academic hospital, where both elective and emergency surgical procedures are performed. Approximately 400 new patients with colorectal cancer are managed each year.

**Figure 1:**
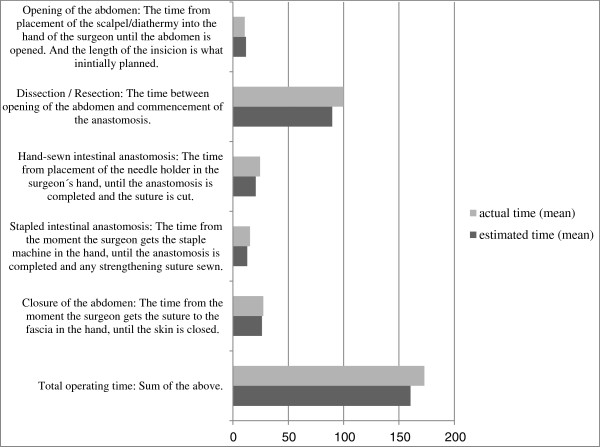
**Definition and time required to perform specified surgical phases of a colon cancer resection.** Estimated time for 18 surgeons’ preoperative self-estimation of specified surgical phases compared with actual time needed to perform the same phases in 21 elective, non-advanced colon cancer resections. Mean values and definitions of phases.

Estimated surgical time was acquired through structured interviews. To avoid selection bias, we contacted all surgeons employed at the hospital’s colorectal department who were consultants or registrars in surgery. Of 19 surgeons asked via email to be interviewed, 18 agreed to participate in the study. One surgeon was not able to participate due to time constraints. Physicians who were receiving their basic surgical training at the colorectal department when the study was conducted were informed about the study by email, as they could possibly be involved in timing the surgical phases.The interviews were conducted at the hospital over two weeks in April 2012. The surgeon chose the time and place, and the interviews lasted 4–30 minutes. The question asked was “how long do you think it generally takes to perform the following surgical phases when performing a resection of the colon?” The operation was divided into five defined phases, and the answers were given in minutes and seconds (Figure 1).

The actual surgical times of 21 resections of the colon, limited to open surgery and elective colon cancer resections with a primary anastomosis, were measured during a six-week period in April–June 2012. Only non-advanced (not involving other organs) cancer resections were included. Before the skin incision was made, when time-out according to the World Health Organization (WHO) surgical safety checklist [7] was performed, the operating room nurse reminded the team that the surgical phases would be timed. The time required for each surgical step was noted in minutes and seconds on a questionnaire by the assistant nurse, who also filled in the International Classification of Diseases (ICD) code, body mass index (BMI), and year of birth of the patient undergoing surgery. In every OT in the investigated operating ward, there is a clock on the wall that is visible, with some effort, to everyone in the room. However, the surgeons were specifically asked not to change anything in their operative approach on account of the time measurements. Of the 22 procedures that were timed, one procedure was excluded due to missing information, resulting in 21 analysed questionnaires.

Ethical approval of the study was granted by the University of Gothenburg, as this survey was carried out as a Master Degree Project in Nursing at the Institute of Health and Care Sciences. Identities of the patients were not noted on the questionnaires used in the OTs.

### Statistical analysis

An independent *t*-test and Mann–Whitney *U* test were used to determine differences in estimated and actual times. All tests were two-tailed. The significance level was set at p = 0.05, and the confidence interval (CI) was 95%. Pearson’s correlation test was used to analyse the relationships between different variables. To avoid mass significance, only the variables shown in Table 1 were selected for correlation analysis.

**Table 1:** Surgical phases and factors affecting a surgical procedure in time

	**r**	**p**	**r**^**2**^
**Opening of the abdomen – Closure of the abdomen**	0.301	0.184	0.09
**Dissection/resection – Closure of the abdomen**	0.464	0.039*	0.21
**Dissection/resection – Hand-sewn intestinal anastomosis + Closure of the abdomen**	0.128	0.602	0.17
**Hand-sewn intestinal anastomosis – closure of the abdomen**	0.536	0.018*	0.29
**BMI – Hand-sewn intestinal anastomosis + Closure of the abdomen**	0.284	0.238	0.81
**BMI – total time for surgical procedure**	0.202	0.380	0.04
**Age – total time for surgical procedure**	0.138	0.551	0.20

Actual time needed to perform specified surgical phases in 21 elective, non-advanced colon cancer resections explored with correlation analysis between surgical phases and also between those phases and patient specific factors.

*Correlation was considered significant at the 0.05 level.

IBM SPSS Statistics, version 20 (SPSS, an IBM Corporation, Somers, NY) was used for all statistical analyses.

## Results

The surgeons who were interviewed responded to all of the questions. The range of time estimates given by the surgeons is illustrated in Figure 1 and Table 2. The greatest range in estimated time was in the time for dissection/resection and total surgical time.

**Table 2:** Estimated and actual surgical time

	**Estimated time n = 18**					**Actual surgical time n = 21**					
	**Median**	**IQR**	**Mean**	**SD**	**Range**	**Median**	**IQR**	**Mean**	**SD**	**Range**	p*
**Opening of the abdomen**	10.0	9.5-15.0	11.8	3.6	7.0-20.0	10.0	6.5-12.0	10.8	5.5	4.0-26.0	0,485
**Dissection/Resection**	87.5	67.5-108.8	89.7	31.1	50.0-180.0	75.0	64.0–126.5	100.0	62.8	43.0-308.0	0,532
**Hand-sewn intestinal** **anastomosis**	20.0	15.0-25.0	20.6	5.6	15.0-35.0	23.0	16.0-27.0	24.5	11.3	11.0-56.0	0,193
**Stapled intestinal** **anastomosis**	10.0	7.8-15.0	12.1	6.4	3.0-30.0	15.5	10.0-15.5	15.5	7.8	10.0-21.0	0,486
**Closure of the abdomen**	25.0	20.0-30.0	26.2	11.1	15.0-60.0	27.0	19.0-34.5	27.5	8.5	14.0-44.0	0,693
**Total time for surgical procedure**	147.0	131.5-201.3	160.4	42.3	110.0-260.0	146.0	128.0-201.0	173.0	72.0	96.0-404.0	0,539

Estimated time for 18 surgeons’ preoperative self-estimation of specified surgical phases compared with actual time needed to perform the same phases in 21 elective, non-advanced colon cancer resections.

*independent *t*-test.

IQR = Inter Quartile Range.

SD = Standard Deviation.

The age range of the included patients was 24–86 years (median = 71; interquartile range (IQR) = 63.5–80.5; mean = 70.1; SD = 14.1). The range of BMIs of the patients was 19.6–34.0 (median = 25.8; IQR = 24.3–28.0; mean = 26.2; SD = 3.3). The following surgical procedures were performed; nine right hemicolectomies, five resections of the sigmoid colon, three left hemicolectomies, two extended resections of the right colon (including resection of the transverse colon), one subtotal colectomy, and one ileocaecal resection. The 21 surgical procedures were distributed among 12 surgeons—three were consultants, four were registrars in surgery, and five were in surgical training.

The differences between estimated and actual time are shown in Table 2 and Figure 1. At a group level, there were no significant differences between mean estimated and actual times for specific surgical phases or total surgical time (160.4 versus 173.0 minutes; p = 0.539 by independent *t*-test; p = 0.921 by Mann–Whitney *U* test).

Variations in actual time are noted in Table 2. As with the time estimates, variations were found mainly in the dissection/resection phase and total surgical procedural time.

As shown in Table 1, there was a positive significant correlation between time needed for dissection/resection and time needed to close the abdomen (r = 0.464, p = 0.039), and between time needed for hand-sewn anastomosis and time needed to close the abdomen (r = 0.536, p = 0.018). In contrast, no statistically significant correlations could be found between BMI and total surgical time (r = 0.046; p = 0.841) or between age and total surgical time (r = 0.09, p = 0.698). There was also no correlation between time needed to open the abdomen and time needed to close the abdomen.

## Discussion

The findings in this survey indicate that it is not beneficial to ask an individual surgeon how much time a surgical procedure will take to complete or how much time is needed for defined phases of a procedure. However, surgeons’ time estimates on a group level correlated well with the actual measured time for specified surgical phases and total surgical procedural time. There was a correlation between the time taken for specific surgical phases and the remaining time of a surgical procedure. The main variation in time was found in the dissection/resection phase.

Surgical procedural time will always vary, depending on the procedure, the patient, the surgeon, the scrub nurse, and the rest of the team in the OT. Both complexity of the surgical procedure and the operating team affect procedural time [8]. Our survey indicates that there are relationships between variables that may be of clinical relevance to surgical teams regarding intraoperative planning and coordination of upcoming surgical procedures. For planning of the continuous surgical day schedule, actual time of the last phases of an operation—hand-sewn anastomosis and closure of the abdomen—are the most important factors. Correlation analysis showed that the longer it took for dissection/resection and hand-sewn anastomosis, the longer it took to close the abdomen. One conclusion might be that after a long and probably difficult surgical procedure, the level of fatigue experienced by the surgeon might result in more time needed for the last phase, which is closure of the abdomen. Another plausible conclusion is that a single surgeon who requires more time than average to perform a hand-sewn anastomosis is likely to require more than the average time to close the abdomen as well.

The main variation in actual time was seen in the dissection/resection phase. This finding can be partially explained by an outlier (dissection/resection time of 308 minutes) that was included in the analysis. That relatively long surgery involved intra-abdominal adhesions, which is not an uncommon occurrence at the investigated department.

Operating surgeons often become involved in changes in priorities in the surgical schedule. A single surgeon is expected to be able to estimate the remaining procedural time when asked during surgery, the intraoperative interruption can be disturbing [9]. In addition, interruptions during surgery can lead to a loss of concentration and affect patient safety [4,10,11]. Knowing the actual surgical time required could lead to a reduced number of intraoperative interruptions and help scrub nurses plan intraoperative care and the final phases of the procedure [12].

With knowledge of the widespread range in estimated time, it is of importance and clinical relevance to obtain actual times for specific phases and to ascertain the factors affecting the length of a surgical procedure. This new awareness could make it easier for all personnel in the OT to estimate remaining procedural time. This knowledge would also likely increase patient safety by providing an instrument to facilitate communication and teamwork in the OT, as communication shortcomings in the OT can affect the result of a surgical procedure [6,12,13]. In addition, knowing the actual times required could simplify communication with the operating ward coordinator in terms of planning the continuous surgical day schedule and coordinating breaks and personnel changes. Furthermore, the ability to schedule the surgical day more accurately in advance should lead to fewer instances of opening the doors to the OT, which in turn increases patient safety. Intraoperative disruptions lead to longer surgical times, and door openings increase the number of bacteria in the air, thus increasing the risk of surgical site infection [14-16].

Surgical procedures today are often technically difficult and require a very high quality of teamwork to ensure that patient safety is not compromised. The modern OT is a high-risk work environment with its high technology, complexity, and potential for patient harm and adverse events [1,17]. A benefit of improved team awareness of the actual surgical time required for specific phases of an operation is that the OT team can more easily determine when a procedure is starting to take longer than usual. For the surgeon, this knowledge would make it easier to know when to request help from a senior colleague or when to take a pause due to the long operation. Engelmann et al. [18] showed that work breaks during complex laparoscopic surgery can reduce a surgeon’s stress level without prolonging the operation.

Although we could not find any previous studies regarding time required to perform specific surgical phases of colorectal cancer resection, Strum et al. [2] identified several factors that affect the time needed to conduct a surgical procedure. The main source of variability in surgical procedural time was surgeon effect; type of anaesthesia, age, gender, and American Society of Anaesthesiologists (ASA) classification were additional factors. Strum et al. argued that the costs of surgical procedures could be reduced through improved scheduling of surgical resources [2], which could also be true for the investigated department. In addition, cancelled procedures lead to the use of unnecessary resources and costs to society, as it is inconvenient and might contribute to increased morbidity of the affected patients [19].

This study has a number of limitations, mostly related to the fact that it is a pilot study with a small sample size. A power calculation based on the results of this survey showed that with a 0.05 significance level and 80% power, we would need to recruit 25 surgeons to be interviewed and 89 procedures to reveal a clinical relevant difference of 30 minutes between estimated and actual procedural time. It should be possible to conduct a study of a similar size in the investigated department in less than a year. Although the sample size of this survey is small, it can be regarded as a strength that 18 of 19 invited surgeons participated in the interviews. Moreover, only one of the 22 included surgical procedures was excluded. With the intent of avoiding selection bias [20] all consultants and registrars and all open surgery resections of the colon with an anastomosis were included. Another weakness is that the interviews were not conducted under the same conditions. As much as possible, the interviews were carried out in private, with as few distractions as possible, and modified to accommodate the often-busy surgeon to be interviewed. Both interviews and time measurement were conducted in a contemporary period. Although the external validity of a single-centre study can always be debated, the concept, ideas, and main findings of this survey should be widely applicable and easy to reproduce in different settings. The small number of time measurements that were performed in this study does not really permit any firm conclusions regarding how much time it takes to perform specific surgical phases or what factors affect that time. This study was designed to examine whether a full-scale study is possible to implement in a similar way.

Ideas for future studies have emerged through this survey. For purposes of coordinating and planning the surgical day schedule, it would be important to know the factors that affect the time requirement before the patient arrives in the OT, as well as the factors that affect the time after the surgical procedure. Likewise, it would be of interest to study the correlation between length of the incision in the fascia and time taken to close the abdomen. Furthermore, it would be of relevance to study the correlation between surgeons’ years of experience and time needed for different phases of a surgical procedure. It would also be of benefit to undertake a similar survey with laparoscopic colon cancer resections. Knowing which phases take longer to perform could help in knowing where to improve operational efficiency. Based on the experiences of this survey, this topic will be investigated further in full-scale studies.

## Conclusions

It can be difficult for a single surgeon to estimate surgical procedural time for a partial or full procedure. This survey has given us an instrument with which to determine actual operating time for specified surgical phases. If every member of the operating team has knowledge of the time needed to perform specific parts of a colon cancer resection, teamwork in the OT is likely to improve. Knowing actual surgical times can provide the operating team with important knowledge to facilitate intraoperative communication. Factors affecting surgical time and actual times of surgical phases would be good instruments to use when planning and scheduling the day’s surgery schedule. A larger study is likely to provide additional time estimates and identify variables that affect surgical time.

## Abbreviations

WHO: World Health Organization; ICD code: International Classification of Diseases; BMI: Body Mass Index; CI: Confidence interval; IQR: Interquartile range; ASA: American society of anesthesiologists classification.

## Competing interests

The authors declare that they have no competing interests.

## Authors’ contribution

Research design: All authors. Acquisition of data: SE. Analysis and interpretation of data: All authors. Drafting the paper: SE, KK. Revising the paper critically: All authors. Approving the submitted version: All authors.
